# Assessment of *Talaromyces Marneffei* Infection of the Intestine in Three Patients and a Systematic Review of Case Reports

**DOI:** 10.1093/ofid/ofaa128

**Published:** 2020-04-27

**Authors:** Mianluan Pan, Jie Huang, Ye Qiu, Wen Zeng, Zhangcheng Li, Shudan Tang, Xuan Wei, Jianquan Zhang

**Affiliations:** 1 Department of Respiratory and Critical Care Medicine, The Eighth Affiliated Hospital, Sun Yat-Sen University, Shenzhen, Guangdong, China; 2 Department of Respiratory and Critical Care Medicine, The First Affiliated Hospital of Guangxi Medical University, Nanning, Guangxi, China; 3 Department of Tuberculosis, Fourth People Hospital of Nanning City, Nanning, Guangxi, China; 4 Department of Comprehensive Internal Medicine, The Affiliated Tumor Hospital of Guangxi Medical University, Nanning, Guangxi, China

**Keywords:** endoscopy, intestinal, stool culture, *Talaromyces marneffei*

## Abstract

**Background:**

Hematogenous dissemination of *Talaromyces marneffei* can result in multiorgan involvement (skin, lung, and reticuloendothelial system involvement); however, few studies have reported intestinal *T marneffei* infections. We investigated clinical features, management, and patient outcomes concerning *Talaromyces*-related intestinal infections.

**Methods:**

Patients with *Talaromycosis* between August 2012 and April 2019 at The First Affiliated Hospital of Guangxi Medical University, China, were retrospectively analyzed. Patients presenting with intestinal *Talaromycosis* and endoscopy-confirmed diagnoses were investigated. We also undertook a systematic review of the relevant English and Chinese literature.

**Results:**

Of 175 patients diagnosed with *Talaromycosis*, 33 presented with gastrointestinal symptoms, and 31 underwent stool cultures, 1 of which tested positive. Three patients had gastrointestinal symptoms and negative stool cultures, and endoscopic tissue biopsy confirmed a pathological diagnosis. A systematic review of 14 reports on human *Talaromycosis* identified an additional 16 patients. Fever, weight loss, and anemia were the most common symptoms, along with abdominal pain, diarrhea, and bloody stools. Abdominal computed tomography showed intestinal wall edema and thickening and/or abdominal lymphadenopathy. Endoscopy showed erosion, hyperemia, edema, and multiple intestinal mucosal ulcers. Of the 19 patients, 16 received antifungal therapy, 14 of whom recovered and 2 died. Three patients received no therapy and died.

**Conclusions:**

Gastrointestinal disseminated *Talaromycosis* is not rare and can affect the stomach, duodenum, and colon, and may involve the entire digestive tract. Colon is the most common site. Endoscopy is needed for patients presenting with gastrointestinal symptoms in *T marneffei-*infected endemic areas. Systemic application of effective antifungal therapy can improve the prognosis.

*Talaromyces marneffei* is an important pathogenic thermally dimorphic fungus that has been reported to cause systemic mycosis in southeast Asia [1, 2]. *Talaromycosis* is endemic in tropical regions, especially Thailand, Vietnam, northeastern regions of India and China, such as in Guangxi, Fujian, Hong Kong, and Taiwan [3]. *Talaromyces marneffei* can cause human infections in both immunocompromised and previously healthy hosts. *Talaromycosis* is categorized as either localized or disseminated. *Talaromyces marneffei* disseminates hematogenous or via the lymphatic system throughout the body, involving the skin, respiratory, digestive, and reticuloendothelial systems [4]. Given the extent of lymphoid tissue throughout the gastrointestinal system, theoretically, it should be a common site of infection. However, intestinal involvement concerning *Talaromycosis* is uncommon, and diagnosis using digestive endoscopy and tissue biopsy culture and pathology is extremely rare. As of March 2020, only 11 cases of intestinal *Talaromycosis* that were diagnosed antemortem by endoscopy have been reported in the literature [5–13]. In this study, we report 3 patients diagnosed with *Talaromycosis* who underwent intestinal tract endoscopy examination, and we conducted a literature search of *Talaromycosis* with gastrointestinal involvement using Chinese and international databases. We describe the clinical features, treatment, patient management, and patient outcomes to provide evidence for early diagnosis and to reduce the possibility of overlooking or misdiagnosing this form of infection.

## METHODS

### Medical Records

We reviewed the medical records of 175 patients who had been diagnosed with *T marneffei* infection between August 2012 and April 2019 at The First Affiliated Hospital of Guangxi Medical University. Among these, 3 patients with gastrointestinal symptoms who had been diagnosed using endoscopy and tissue biopsy pathology were retrospectively evaluated. This study was approved by the Ethics Committee of the Faculty of Medicine, The First Affiliated Hospital of Guangxi Medical University, and all patients provided written informed consent.

### Systematic Review

A literature search for *Talaromycosis*-related gastrointestinal involvement was undertaken using the Public Medline (PubMed) database, the China National Knowledge Infrastructure (CNKI) database (http://www.cnki.net), the VIP Database for Chinese Technical Periodicals (CQVIP) (http://lib.cqvip.com/), and the Wanfang database (http://g.wanfangdata.com.cn/). Reports published between 1970 and March 2020 in Chinese and English were reviewed. The key words searched were “*Penicillium marneffei*,” “*Penicilliosis*,” “*Talaromyces marneffei*,” and “*Talaromycosis*.” References cited in the retrieved articles were reviewed for relevant citations. All article abstracts were reviewed, and full-text versions of relevant articles were retrieved for data extraction and analysis.

### Definition of *Talaromyces marneffei* Local Gastrointestinal System Infection

*Talaromyces marneffei* gastrointestinal system infections were defined as local or disseminated, and they included the upper mouth, pharynx, esophagus, stomach, and small intestine and/or the lower digestive tract (jejunum, ileum, and large intestine).

### Inclusion and Exclusion Criteria

Inclusion criteria comprised patients with *T marneffei* infection and intestinal involvement that had been (1) surgically confirmed or (2) confirmed on autopsy or using endoscopic biopsy tissue samples and pathology and culture examinations. Exclusion criteria comprised patients who presented with gastrointestinal symptoms but who had unconfirmed *T marneffei* infection according to histopathology or culture results. For duplicate publications, the most recent article was used for data extraction.

### Data Extraction

Data were extracted and tabulated according to year of publication, patient demographics, clinical presentation, *Talaromycosis* outcome, underlying disease, and human immunodeficiency virus (HIV) status.

## RESULTS

During the 6-year study period, 175 patients presented with histopathology- and/or culture-confirmed *Talaromycosis*; 86 patients (49.14%) were HIV-positive. Among 175 patients, 33 (18.86%) presented with gastrointestinal symptoms (such as abdominal pain [n = 33], diarrhea [n = 26], malaise and vomiting [n = 25], bloating [n = 21], and bloody stools [n = 2]); 31 patients (17.71%) underwent a stool culture; and only 1 culture (3.23%) was positive. Only 3 patients (1.71%) had a pathological diagnosis made using endoscopic tissue biopsy samples confirm intestine *Talaromycosis*, but their stool cultures were negative.

## CASE REPORTS

### Patient 1

**Figure 1.**
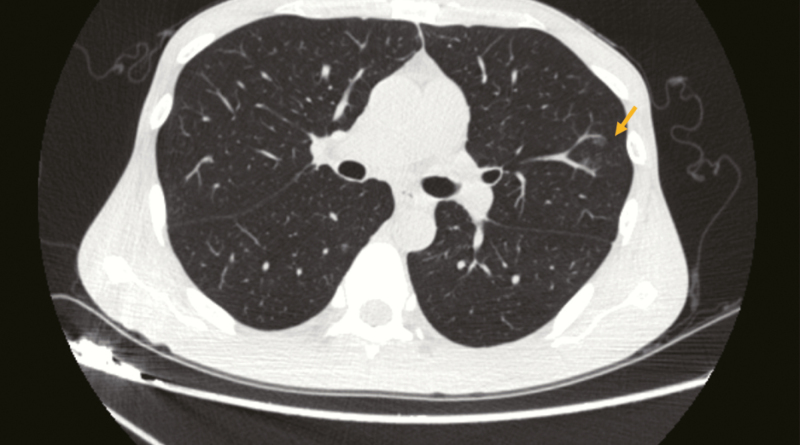
The chest CT manifestations of patient 1 showing plaques and exudation.

**Figure 2.**
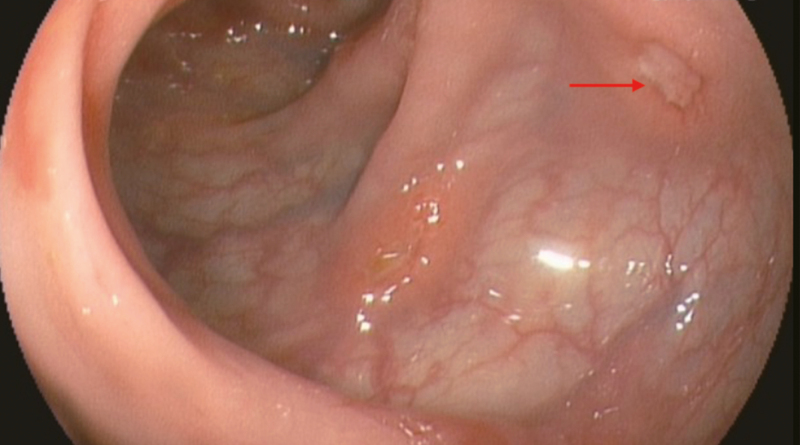
Colonoscopy showed a shallow ulcer (arrow).

**Figure 3.**
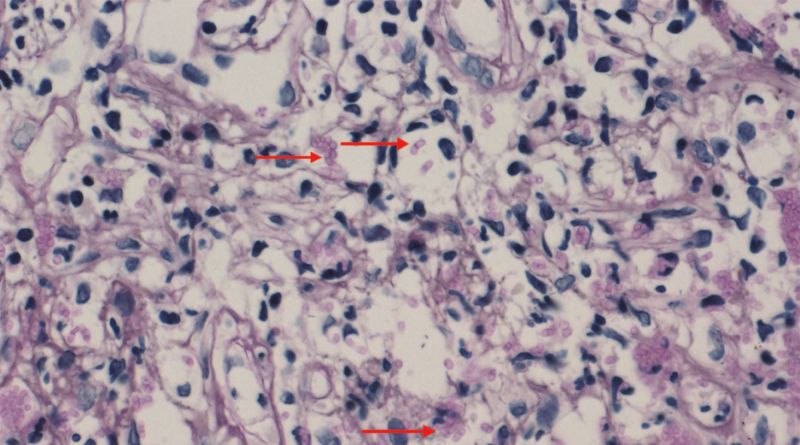
Microscopic appearance of tissues revealed separated yeast (arrow) inside histiocytes (D-periodic acid-Schiff), a finding characteristic of *T marneffei* (magnification × 400).

**Figure 4.**
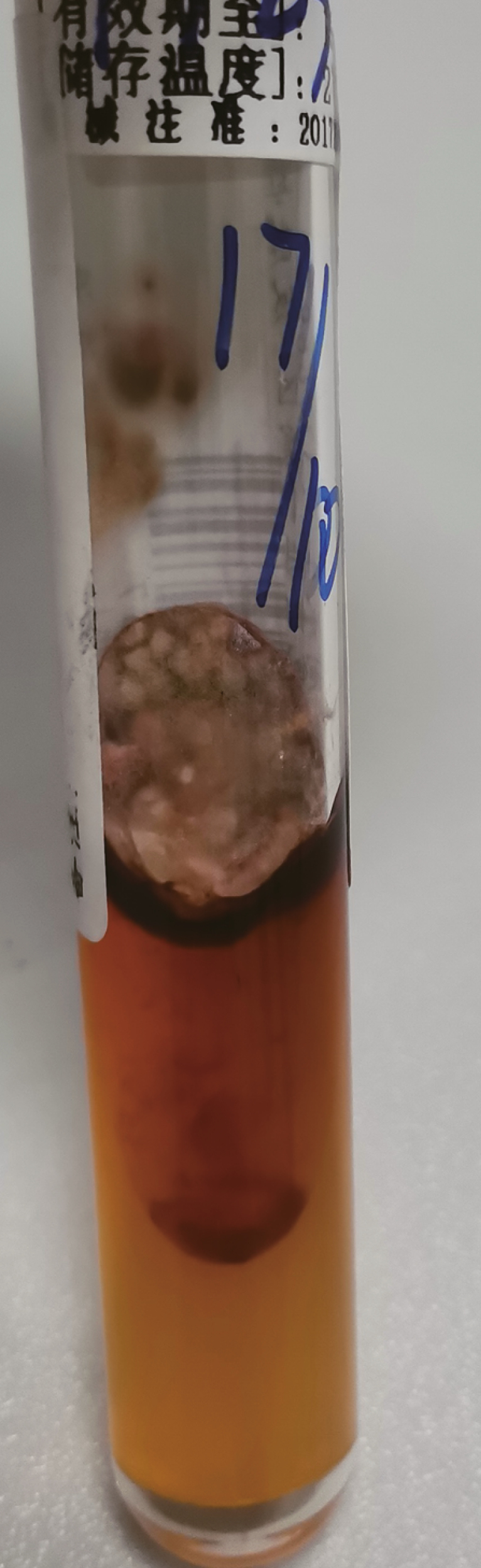
At 25˚C on Sabouraud dextrose agar (SDA), the mold from cultured blood demonstrating white to tan-colored, velvety, flat colonies with a red soluble pigment.

A 37-year-old man was admitted to hospital with a >2-week history of abdominal pain and diarrhea, and a 7-kg weight loss. He had previously been in good health and did not report any known exposure to bamboo rats. On physical examination, his temperature was 38.5°C, and enlarged cervical and inguinal lymph nodes were observed. An abdominal examination revealed his liver was palpable 4 cm below his respective costal margins, whereas the remainder of the physical examinations were unremarkable. Routine blood test results revealed a decreased leukocyte count (2.13 × 10^9^/L); neutrophil count (1.46 × 10^9^/L); lymphocyte count (0.44 × 10^9^/L); and hemoglobin level (9.8 g/dL). The erythrocyte sedimentation rate (ESR) was 42 mm/hour. The stool culture was negative, and elevated the serum beta-d-glucan (G-test) measurement was 783.80 pg/mL. Abnormal biochemistry test showed low albumin levels and elevated transaminase levels. The results were as follows: albumin, 23.6 g/L; aspartate, 112 U/L; and alanine aminotransferase, 84 U/L. Serum immunoglobulin (Ig)G, IgA, and IgE levels were elevated, whereas IgM level was decreased. Natural killer cell percentage was 7.97% (normal 9%–15%). The CD4^+^ and CD8^+^ T-lymphocyte counts were 77 and 397 cells/μL, respectively. A test for antibodies to HIV-1 was positive. Liver B-mode ultrasound imaging showed that the right upper boundary of the liver was located in the 6th intercostal space. Chest computed tomography (CT) showed plaques, nodules, and exudation disseminated throughout both lung fields, especially in the upper left lung (Figure 1). A colonoscopy revealed a scattered annular ulcer and circular protuberance erosion, from the ileocecum to the rectum, with healthy mucosa observed between the lesions (Figure 2). A biopsy of ileocecal tissue ulcers showed cell proliferation in the lamina propria and fungal yeast in the cytoplasm. D-periodic acid-Schiff (PAS) staining showed central septation of yeasts consistent with *T marneffei* (Figure 3). A blood culture at 25℃ and 37℃ on Sabouraud dextrose agar (SDA) subsequently confirmed *T marneffei* (Figure 4). The patient’s final diagnosis was disseminated *Talaromycosis* involving the lung, liver, colon, blood, and lymph nodes. The patient was prescribed parenteral amphotericin B (1 mg/kg for 2 weeks). His abdominal pain resolved promptly after 1 week, and he was maintained on oral itraconazole at a daily dose of 400 mg combined with highly active antiretroviral therapy and reported no recurrence of his symptoms at 8 months follow-up.

### Patient 2

**Figure 5.**
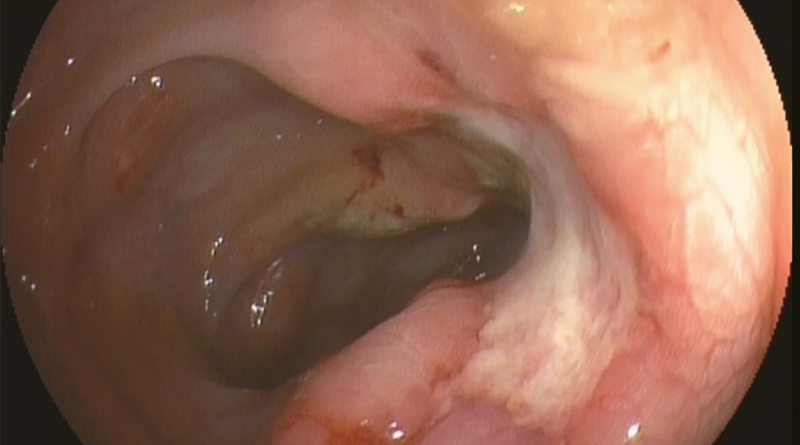
Colonoscopy showed a large ulcer, uneven bottom, nodular protuberance of peripheral mucosa.

**Figure 6.**
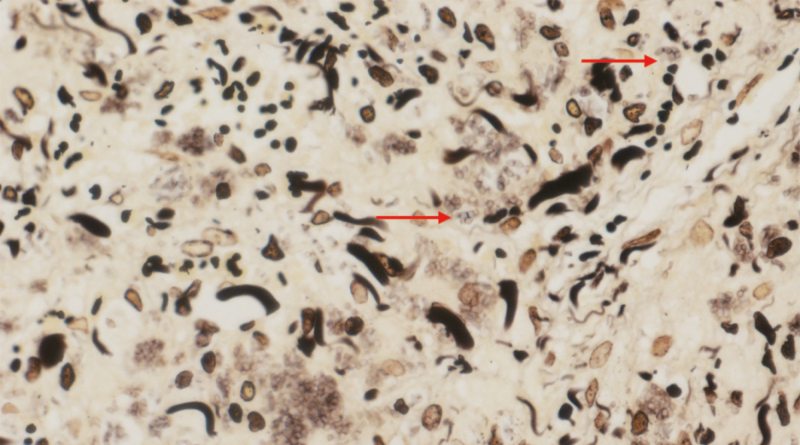
Grocott methenamine silver staining of tissue showed several yeast-like organisms with cells ranging from 2 to 3 μm in diameter, and septate forms (arrow) (magnification × 400).

A 50-year-old man presented with a 2-month history of abdominal pain before admission and a 6-kg weight loss. On physical examination, superficial lymph nodes were nonpalpable. Abdominal examination indicated no enlargement of the liver or spleen, and the physical examination was otherwise unremarkable. The patient’s hemoglobin level was 9.1 g/dL, the lymphocyte count was normal, and the serum albumin level was 31.5 g/L. The CD4^+^ and CD8^+^ T-lymphocyte counts were 110 and 1095 cells/μL, respectively. The C-reactive protein (CRP) level was 20.68 mg/L, and the ESR was 55 mm/hour. His stool sample contained blood, but the culture results were negative. An HIV serology test was positive. Colonoscopy showed ulcers of different sizes located from the rectum to the cecum. The largest ulcer (approximately 3 cm in length) was located near the liver curve of the transverse colon and had an uneven bottom, nodular protuberance of the peripheral mucosa. Healthy mucosa was observed between the ulcers (Figure 5). Grocott methenamine silver stain for histopathologic evaluation of biopsies derived from the transverse colon tissue showed several yeast-like organisms with red cells ranging from 2 to 3 μm in diameter (Figure 6). These yeast-like organisms were spherical to oval in shape and had a transverse septum, and a definitive diagnosis of *Talaromycosis* involving the colon was confirmed. The patient was prescribed parenteral fluconazole with amphotericin B (0.5 mg/kg for 2 weeks), and his abdominal pain improved. He was then prescribed itraconazole (200 mg twice a day) and started on highly active antiretroviral therapy. At his 1-year follow-up consultation, he had remained asymptomatic.

### Patient 3

**Figure 7.**
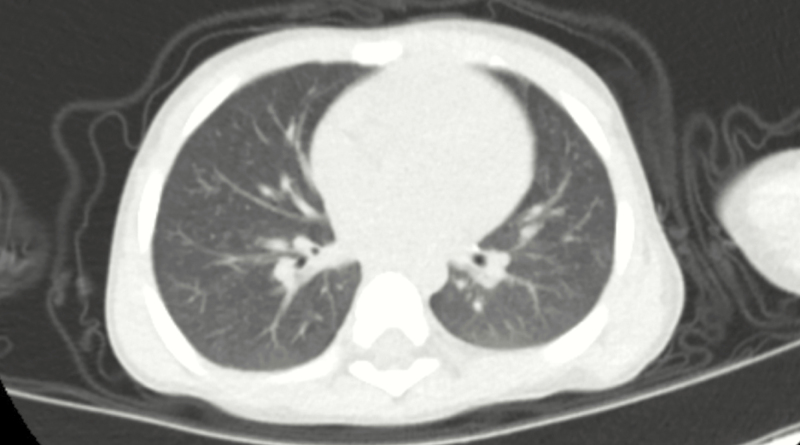
The chest CT manifestations of patient 3 showing plaques.

**Figure 8.**
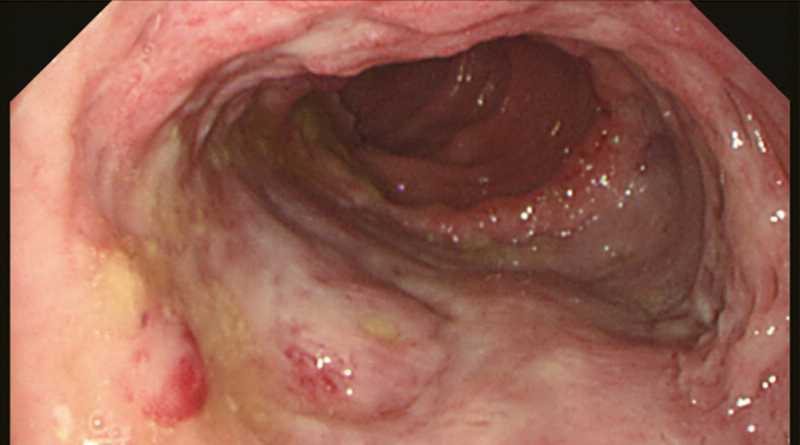
Colonoscopy showed the mucosa was obviously hyperemia and edema, showing cobblestone pattern change, with scattered erosion and ulcer.

**Figure 9.**
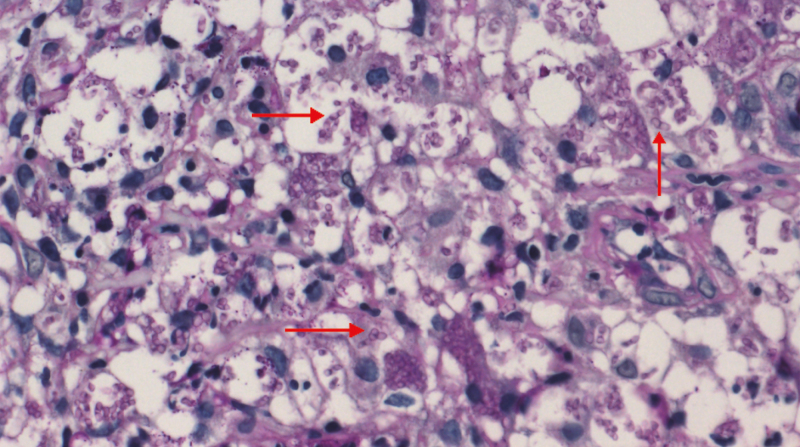
PAS staining of colon ulcers tissue showed revealed some intracellular and extracellular yeasts, elongated, and septate forms (arrow) (magnification × 400).

A 37-month-old boy with a history of recurrent pneumonia since infancy exhibited recurrent pain in the right abdomen and fever for 3 months. The abdominal pain was related to eating food but not to physical activity and position. His maximum temperature was 40℃. Physical examination revealed lymphadenopathy in the left neck region. The liver and spleen were palpable 4 cm below his respective costal margins. Routine blood examination revealed an elevated leukocyte count at 16.9 × 10^9^/L, neutrophil count at 9.5 × 10^9^/L, and lymphocyte count at 4.4 × 10^9^/L and decreased hemoglobin level at 9.0 g/dL. Blood was present in the stool sample. His serum albumin level was 29.0 g/L, the CRP level was >192 mg/L, and the ESR was 28 mm/hour. The aspergillus galactomannan antigen was 0.826 (normal, <0.5). Serum IgM level was slightly elevated, whereas IgG and IgA levels were normal. Antinuclear and anti-HIV antibodies as well as the interferon-γ autoantibody were negative, as were his blood and stool cultures. Chest CT showed plaques disseminated throughout the left lower lung and the upper lobe of the right lung (Figure 7). Contrast-enhanced abdominal CT showed hepatomegaly and intestinal wall thickening in the ascending colon and cecum, with mesenteric lymphadenopathy. Subsequently, he underwent an ultrasound-guided liver puncture. The colonoscopy showed a cobblestone pattern (nonulcerated mucosa, separated by eroding ulcers, ulcers, polypoid lesions, and lumen deformation) ranging from the colon to the cecum (Figure 8). *Talaromyces marneffei* was isolated from the mucous membrane of the colon as well as the liver tissue (Figure 9). A bone marrow culture at 25℃ and 37℃ on SDA also confirmed *T marneffei* after 2 weeks. A diagnosis of disseminated *Talaromycosis* involving the liver, colon, lymph nodes, and bone marrow was made. Intravenous voriconazole (12 mg/kg once every 12 hours) was administered for 4 weeks. Subsequently, the liver volume reduced and was palpable 1 cm below his respective costal margins, and the leukocyte and neutrophil counts returned to normal. The medication was then changed to oral voriconazole (7 mg/kg twice a day); however, abdominal pain and fever recurred. Abdominal plain radiography revealed a bowel perforation, pneumoperitoneum, and intestinal obstruction. Hence, an emergency exploratory laparotomy with intestinal resection, anastomosis, and colostomy was performed. Postoperative pathology indicated the presence of *T marneffei.* The patient’s condition improved after intravenous voriconazole administration and subsequent oral voriconazole for 6 months. A review colonoscopy showed good recovery of the stoma, which was located at the ileocecum, within 35 cm from the anal verge. No relapse was observed during the 18-month period of antifungal treatment.

### Systematic Review

**Figure 10.**
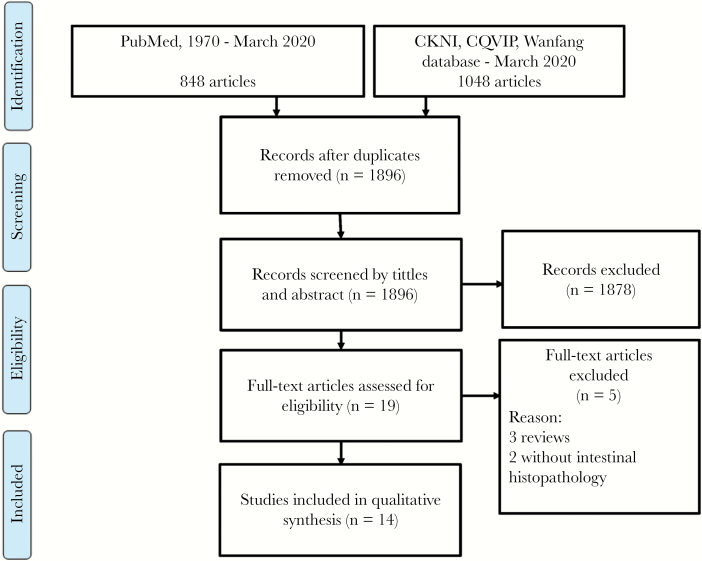
PRISMA (2009) Flow diagram. CNKI, China National Knowledge Infrastructure; CQVIP, VIP Database for Chinese Technical Periodicals.

The search process and result are illustrated by a flowchart as shown in Figure 10. Ten English and 4 Chinese articles (involving 16 patients) were identified, in addition to the 3 patients we report, with confirmed *Talaromycosis* gastrointestinal involvement. Detailed clinical information concerning these 19 patients are presented in Table 1 [5–18]. Of 17 males and 2 female, 2 patients were Indian, and 17 patients were Chinese residents of mainland China (n = 9), Hong Kong (n = 5), and Taiwan (n = 3), comprising 12 HIV-infected patients and 7 HIV-negative patients. Their median age was 38 years (range, 32–52 years). The common clinical manifestations among all patients included abdomen pain (63.2%), fever (57.9%), weight loss (42.1%), diarrhea (31.5%), and bloody stools (26.3%). Abdominal CT results showed abdominal lymphadenopathy in 6 patients. Endoscopy showed erosion, hyperemia, edema, and multiple ulcers of the intestinal mucosa in 15 patients. *Talaromycosis* has been reported to involve the stomach, duodenum, and colon; and the adjacent mesenteric lymph nodes and liver can also be involved. A positive fungi detection in culture was found in the blood (6 patients), skin lesions (3 patients), bone marrow (3 patients), and duodenal tissue (2 patients), in combination with cytologic or histologic examinations of biopsied colon (15 patients), liver (3 patients), duodenal tissue (2 patients), small intestine(1 patient), and mesenteric lymph nodes (2 patient). However, of 19 patients, 16 received antifungal therapy, 14 of whom recovered and 2 died. In addition, 3 patients did not undergo therapy and died.

**Table 1.** Summary of the Clinical Characteristics Concerning 19 Patients With Gastrointestinal *Talaromycosis*: Systematic Literature and Present Report Review

Year of Publication	Area of Report	Age (Year)/Sex	Occupation	Medical History	Clinical Manifestations	CD4^+^ (cells/ mm^3^)	Endoscopic Findings /Abdomen CT	Site(s) of Positive Culture/Histology	Antifungal Treatment	Outcome
1988 [13]	Hong Kong, China	58/M	NM	Hemolytic anemia	Fever, abdominal pain, anemia, hepatosplenomegaly	ND	Upper descending, colon constriction^a^	Liver, colon, lung (a + h)	AMB for 5 days	Died
1988 [14]	China	0.33/M	None	None	Fever, diarrhea, anemia, hepatosplenomegaly	ND	ND	Liver, spleen, bowel, kidney, lung, adrenal, mesenteric lymph nodes (a + h)	None	Died
1992 [15]	Hong Kong, China	72/M	NM	AIDS	Dysphagia, anorexia, weight loss, GI bleeding	ND	ND	Small intestine, mesenteric lymph node, liver (a + h)	None	Died
1996 [5]	Hong Kong, China	32/M	NM	AIDS, TB	Fever, night sweats, dry cough, diarrhea hepatomegaly	60	Multiple ulcers^a^	Colon ulcer (b + c)	AMB for 2 weeks, oral ICZ	Cured
1999 [6]	Taiwan, China	33/M	NM	Renal transplant	Cough and sputum production, bloody stool, tongue ulcer	NM	Erosion at the antrum, ampulla Vater tumor with bleeding^a^	Duodenum (b), blood (c)	None	Died
1999 [6]	Taiwan, China	52/M	NM	AIDS	Fever, abdominal pain, diarrhea, hepatomegaly	20	Shallow ulcers^a^	Colon ulcers (b), blood, bone marrow, skin lesion (c)	AMB for 2 weeks, oral ICZ	Cured
1999 [6]	Taiwan, China	30/M	NM	AIDS	Fever, dyspepsia, abdominal pain, diarrhea, bloody stool, weight loss	ND	Shallow ulcers^a^; mesenteric lymphadenopathy and edematous intestine^b^	Colon ulcer (b), blood (c)	AMB for 2 weeks, oral ICZ	Cured
2004 [7]	China	21/M	Farmer	Tuberculosis of lymph nodes	Abdominal pain, skin lesion, bloody stools	ND	Erosion at the colon^a^	Skin lesions (b + c), colon (b + h)	Oral ICZ for 10 days	Died
2006 [16]	China	51/M	NM	AIDS	Fever, diarrhea, weight loss, skin lesions	20	Multiple ulcers^a^; abdominal lymphadenopathy^b^	Blood, skin lesion (c)	Oral FLZ for 12 weeks, oral ICZ 10 weeks	Cured
2008 [17]	India	33/M	NM	None	Fever, abdominal pain, vomiting, weight loss hematemesis, anorexia, lymphadenopathy	7	Mesenteric lymphadenopathy and intestinal obstruction^b^	Colon (b), duodenal tissue, bone marrow (c)	AMB for 2 weeks, oral ICZ for 10 weeks	Cured
2010 [8]	Hong Kong, China	39/M	NM	AIDS	Fever, diarrhea, sore throat, weight loss	11	Multiple ulcers^a^	Colon ulcers, stomach and duodenal (b + c)	AMB for 2 weeks, then oral ICZ for 10 weeks	Cured
2015 [9]	Hong Kong, China	56/M	NM	Waldenström macroglobulinemia, ITP, PBC	Fever, night sweating, cough, bloody diarrhea	315	Multiple shallow ulcers^a^	Terminal ileal ulcers, stool (c), nasopharyngeal (b + c)	AMB for 2 weeks, oral VCZ	Cured
2016 [10]	China	41/M	NM	AIDS	Fever, abdominal pain, cough, weight loss	18	Multiple ulcers with polypoid lesions^a^	Colon (b)	AMB for 2 weeks, oral ICZ for 9 months	Cured
2017 [11]	China	32/F	NM	AIDS, HBV carriers	Fever, abdominal pain, diarrhea, lymphadenopathy, weight loss	4	Multiple ulcers^a^ abdominal lymphadenopathy^b^	Colon (b), blood (c)	AMB for 2 weeks, oral ICZ for 3 months	Cured
2017 [12]	China	52/M	NM	AIDS	Abdominal pain, weight loss, anemia, diarrhea	28	Multiple ulcers^a^; mesenteric lymphadenopathy, edem-atous expansion of the colon^b^	transverse colon (b + h)	iv ICZ for 1 week, oral ICZ	Cured
2020 [13]	India	38/F	Teacher	AIDS	Abdominal pain, skin lesions, anorexia, weight loss	69	Deep ulceration, luminal narrowing^a^, intestinal obstruction^b^	Skin lesions, jejunal ulcers (b)	AMB for 2 weeks, oral ICZ for 6 months	Cured
PR	China	37/M	Farmer	AIDS	Abdominal pain	77	Multiple ulcers^a^	Colon (b), blood (c)	AMB for 2 weeks, then oral ICZ for 8 months	Cured
PR	China	50/M	Farmer	AIDS	Abdominal pain, weight loss	110	Multiple ulcers^a^	Colon (b)	FLZ + AMB for 2 weeks, oral ICZ for 12 months	Cured
PR	China	3/M	None	None	Abdominal pain, fever, hepatomegaly	1078	Multiple ulcers^a^	Colon, liver tissue (b), bone marrow (c)	iv VCZ for 4 weeks, oral VCZ for 16 months	Cured

Abbreviations: AIDS, acquired immunodeficiency syndrome; AMB, amphotericin B; CT, computed tomography; FLZ, fluconazole; GI, gastrointestinal; HBV, hepatitis B virus; ICZ, itraconazole; ITP, idiopathic thrombocytopenic purpura; NM, not mentioned; ND, not done; iv, intravenous; PBC, primary biliary cirrhosis; PR, present report; TB, tuberculosis; VCZ, voriconazole.

NOTE: Diagnostic methods to demonstrate *Talaromyces marneffei* involved: (a) autopsy, (b) biopsy, (c) culture, and (h) histopathology.

^a^Endoscopic findings.

^b^CT findings of the abdomen.

## Discussion

*Talaromycosis* is not only a common HIV-associated opportunistic infection but has also increasingly been observed in HIV-negative individuals, which serious harm to human public health. The lungs, skin, lymph nodes, and liver are the most frequently involved sites [1, 19]. A laboratory diagnosis involves microscopy and/or culture to identify the fungus from a variety of clinical specimens. Lymphoid tissue is extensive throughout the digestive tract, yet few reports concerning *Talaromycosis* involving the digestive tract have been published. Furthermore, in clinical practice, *Talaromycosis* involving the digestive tract may be considered to be rare.

In this retrospective analysis, in 33 patients with digestive tract symptoms, only 3 patients were diagnosed with *Talaromycosis*, using endoscopy-guided histopathology of intestinal tissue samples, indicating that *Talaromycosis* involving digestive tract symptoms may be less rare than possibly considered. Abdominal pain, fever, weight loss, diarrhea, and bloody stools were the most common clinical presentations. In our study, 14.9% of patients presented with diarrhea, which is similar to that reported in a review of 150 HIV-positive and HIV-negative patients from Thailand (11.3%) [20]. In addition, diarrhea is more common in HIV-infected gastrointestinal *Talaromycosis*. A definitive diagnosis of *T marneffei* infection according to fungal culture has been reported with 100%, 90%, and 76% sensitivity from bone marrow, skin biopsy, and blood samples, respectively [21]. Intestinal involvement in patients with disseminated *Talaromycosis* might have been underdiagnosed because diagnostic yields concerning blood, bone marrow, and skin cultures are sufficiently high to have rendered endoscopy-guided investigations of the intestinal unnecessary. Clinicians may fail to undertake gastrointestinal endoscopy in time to determine the affected site and the severity of the infection, which may lead to serious complications such as intestinal perforation or intestinal obstruction. Therefore, early diagnosis to improve clinical efficacy is extremely important.

In this study, 31 patients with gastrointestinal symptoms were examined using stool cultures, yet only 1 culture tested positive: a positive stool culture may indicate the presence of digestive tract involvement, but the patient did not undergo an endoscopy examination. Therefore, we were unable to determine the specific location or the severity of *Talaromycosis* involving the digestive tract in this patient. The positive rate of stool culture is low and might have been due to poor samples or less pathogenic fungus in the stools. Another reason might have been that the fungus was ingested via food and invaded the intestine, accumulating in lymphoid tissue of the intestinal wall when the local defense function of intestinal mucosa was reduced, causing intestinal infection and leading, in turn, to a hematogenous or lymphatic system spread of *T marneffei* throughout the body. Therefore, there may have been too few fungi in the intestinal cavity to cultivate a positive result. Moreover, intestinal involvement due to disseminated *Talaromycosis* via a hematogenous or lymph node system may follow infection in other parts of the human body. Therefore, the relevant mechanisms in relation to the low stool culture rates require further research.

The bamboo rat is the natural host of *T marneffei* [22]. Fungi can be inhaled and cause a primary pulmonary infection before dissemination, or they may be ingested and invade the intestine and then spread throughout the body. The 3 patients in our study who resided in an area endemic for *Talaromycosis* did not have a history of bamboo rat contact, and their infection route remains unclear. The decrease of CD4^+^ T-lymphocyte count in the intestinal lymphoid tissue of patients with acquired immune deficiency syndrome (AIDS) may relate to the decrease of CD4^+^ T-lymphocyte count in the blood, leading to reduced local defense function, and then potentially increased risk for intestinal various opportunistic infections [17]. In our systematic review, all the AIDS patients’ CD4^+^ lymphocyte count was below 100 cells/mm^3^. Patient 2 presented with abdominal pain and emaciation but had no skin lesions. A colonoscopy showed multiple ulcers, and *T marneffei* was confirmed using mucosal histopathology samples. It appeared that *Talaromycosis* was limited to the intestinal only. At present, patient 2 is the second reported case of *Talaromycosis* found to be limited to the intestinal [8]. Abdominal CT scans showed thickening of the intestinal wall or narrowing of the lumen and extensive abdominal lymphadenopathy. However, abdominal lymphadenopathy may be misdiagnosed as gastrointestinal lymphoma, leukemia, or Kaposi’s sarcoma, in addition to primary tumor of the intestinal tract. In these circumstances, a diagnosis can be made only through histiocyte examination under oil immersion, with the recognition of characteristic yeast-form cells and with the use of particular stains, and the key means of differentiation involves performing a histopathological biopsy. Moreover, if *Talaromycosis* presents with no gastrointestinal symptoms, but abdominal imaging shows that the intestinal wall is thickened or the abdominal lymph nodes are swollen, an endoscopy should be performed to determine whether there is intestinal infection. When an endoscopy examination shows multiple erosions, ulcers, masses, thickening and edema of the intestinal wall, and a narrow lumen, misdiagnosis of ulcerative colitis, Crohn’s disease, or intestinal tuberculosis may occur. Performing a colonoscopy-guided biopsy had positive diagnostic significance. In our systematic literature review, even when a stool culture was negative, endoscopy showed multiple ulcers of the intestinal mucosa, and, ultimately, a diagnosis of *Talaromycosis* was made using histologic examination of biopsied colon tissue samples. Furthermore, the diagnosis using endoscopy was made earlier than previously possible through relying on conventional microbiologic cultures, which require waiting for culture results from 1 to 2 weeks. Therefore, in patients with *Talaromycosis* and gastrointestinal symptoms, an endoscopy with pathological biopsy and culture should be performed to indicate the specific location affected, and the severity of the infection, and to avoid severe complications such as intestinal obstruction or intestinal perforation.

Disseminated *Talaromycosis* can affect the esophagus, the duodenum, the small intestine, the colon, and even the entire digestive tract, with the colon being the most common site of infection (Table 1). Disseminated *Talaromycosis* has also been reported to be accompanied with adjacent mesenteric lymph nodes and liver involvement [14, 16]. If a patient who has resided in or traveled to southeast Asia presents with gastrointestinal symptoms such as abdominal pain, diarrhea, and bloody stools, clinicians should consider *Talaromycosis* involving the digestive tract, endoscopy examination using histopathology and/or tissue cultures, and appropriate use of special stains to demonstrate the yeast-form cells, to improve the detection rate of *Talaromycosis*.

It has been reported that early and effective antifungal therapy can improve the prognosis of *Talaromycosis* [1]. Amphotericin B, itraconazole, voriconazole, and posaconazole are all sensitive to *T marneffei*, but fluconazole is easily resistant, so it is not recommended as a first treatment choice [23]. Five patients died during the study period, with a mortality rate of 26.3%. Two patients died in the course of treatment because of severe systemic inflammatory responses, and 3 patients died without antifungal therapy. Active and effective systemic antifungal treatment can improve the prognosis. Eleven patients received amphotericin B combined with itraconazole antifungal therapy, with a cure rate of 100%. For HIV-positive patients, antifungal therapy combined with highly active antiretroviral therapy significantly improved the clinical symptoms for patients 1 and 2. It remains challenging to inactivate living bacteria in the diseased intestinal segment, so the disease can still be aggravated during antifungal treatment. Patient 3 received antifungal therapy and showed a significant improvement in symptoms. However, intestinal perforation still occurred >1 month after antifungal treatment and progressed to intestinal perforation. *Talaromycosis* was also confirmed in the final resection of the diseased intestinal segment, indicating that *T marneffei* has high pathogenicity. In cases of severe or lethal infection, it is essential to perform appropriate surgery to remove the gut lesion in a timely fashion. *Talaromyces marneffei* is an endosporal pathogen and, after infection, there is cellular immune dysfunction, the inherent immune response of macrophages is inhibited, and the apoptosis of macrophages is reduced. Therefore, for patients with immunodeficiency, longer-term secondary prophylaxis and follow-up may be necessary. We have reported that patients may still relapse after being treated for 4 years [24]. A decision to stop treatment should be comprehensively evaluated according to patients’ clinical symptoms and laboratory test results.

## Conclusions

Disseminated *Talaromycosis* involving the gastrointestinal system is less rare than may have been thought and can include the colon (the most common site), duodenum, and stomach, along with the liver and mesenteric lymph nodes. Given the limited specificity in terms of its clinical manifestations, this form of infection may be easily misdiagnosed or overlooked. In *T marneffei*-endemic areas, where *Talaromycosis* presents with gastrointestinal symptoms, endoscopy should be performed to determine intestinal involvement. Performance of endoscopy to obtain tissue specimens for histopathological analysis and culture might improve the detection rate of *T marneffei* invading the digestive tract. After diagnosis, systemic application of effective antifungal therapy might improve the prognosis. More attention should be paid to the mechanism of *T marneffei* infection in the gastrointestinal tract.
